# Burden of non-alcoholic steatohepatitis among children with type 2 diabetes mellitus

**DOI:** 10.1186/s13098-021-00665-0

**Published:** 2021-04-21

**Authors:** Lauren Parlett, Qinli Ma, Qian Shi, Geoffrey Crawford, Laura Herrera Scott, Vincent Willey, Abiy Agiro

**Affiliations:** 1 HealthCore, Inc, Wilmington, DE USA; 2 Anthem, Inc, Indianapolis, IN USA

**Keywords:** Non-alcoholic steatohepatitis, Type 2 diabetes mellitus, Children, Healthcare utilization and cost

## Abstract

This claims-based retrospective cohort study examined the prevalence and incremental impact of non-alcoholic steatohepatitis among children with type 2 diabetes mellitus in the United States. Although diagnoses of non-alcoholic steatohepatitis were not common among diabetic children, it was associated with significantly higher incremental healthcare cost and risk of hospitalization.

## Introduction

Non-alcoholic steatohepatitis (NASH) and type 2 diabetes mellitus (T2DM) share insulin resistance and hyperinsulinemia from a physiologic perspective. Over the past two decades, clinicians have reported an increasing prevalence of comorbid childhood NASH and T2DM [1]. Prevalence of T2DM is estimated to be 11% among children with a definite NASH diagnoses (biopsy-verified) and 7% among children with borderline or definite NASH [1]. However, prevalence and incremental impact of NASH among children with T2DM are less known.

## Methods

This retrospective cohort study characterized members under the age of 18 with NASH and T2DM (with or without NASH) using a large, geographically diverse, longitudinal database of medical and pharmacy claims from commercial plans in 14 states and Medicaid claims in 22 states in the United States. We examined all children with a claims-based diagnosis of NASH and/or T2DM between October 1, 2015 and October 31, 2017 who had at least 12 months of continuous health plan enrollment prior to diagnosis.

Follow-up occurred until the end of the study period (October 31, 2018), end of insurance enrollment, death, or the occurrence of a hepatic-related outcome (cirrhosis, hepatocellular carcinoma, liver transplant). Follow-up univariate and multivariable analyses assessing cost and healthcare utilization were performed on a greedy nearest neighbor propensity score-matched 1:1 study sample consisting of NASH + T2DM and T2DM only groups. Variables used for propensity score matching are listed in Footnote A in Table 2. The study did not require institutional review board review as it was not considered human subject research and only de-identified data were used.

## Results

Data in Table 1 show different co-occurrence estimates, depending on the base population. Almost 10% (50/519) of children with NASH had T2DM and 0.6% (50/8124) of children with T2DM had NASH. Metabolic syndrome baseline comorbidities revealed that children diagnosed with NASH + T2DM presented similar to children with NASH only (higher prevalence of obesity and dyslipidemia) as compared to children with T2DM only (with the exception of systemic hypertension). Compared to T2DM only, members with NASH + T2DM have more than three times the odds of systemic hypertension (Odds Ratio = 3.51; 95% confidence interval (CI) 1.88, 6.53). The hepatic outcomes (e.g., cirrhosis) occurred rarely, mainly observed on the T2DM only group, and totaled fewer than our masking threshold of 10.

**Table 1:** Demographic and clinical characteristics of children (commercial and Medicaid) with NASH, T2DM or both during 12 month baseline period (before matching)

	NASH only^a,b^	NASH + T2DM^a,b^	T2DM only^a^	Odds Ratio (95% CI) NASH + T2DM compared to NASH only^c^	Odds Ratio (95% CI) NASH + T2DM compared to T2DM only^c^
Sample size, N	469	50	8074		
Female, n (%)	166 (35)	29 (58)	4355 (54)	**2.52 (1.39−4.56)**	**1.18 (0.67−2.07)**
Age, mean (SD)	12.4 (3.2)	14.5 (2.5)	12.6 (4.0)	–	–
Age, median (IQR)	13 (5)	15 (2)	14 (5)	–	–
Age category, n (%)					
0–2	≤ 10	≤ 10	82 (1)	–	–
3–4	≤ 10	≤ 10	369 (5)	–	–
5–11	177 (38)	≤ 10	2069 (26)	–	–
12–17	286 (61)	44 (88)	5554 (69)	**4.69 (1.96−11.23)**	**3.33 (1.42−7.82)**
Health Plan, n (%)					
Medicaid (N = 4514)	353 (75)	36 (72)	4125 (51)	0.85 (0.44−1.62)	**2.46 (1.33−4.57)**
Commercial (N = 4079)	116 (25)	14 (28)	3949 (49)	1.18 (0.62−2.27)	0.41 (0.22−0.75)
Comorbidities at baseline, n (%)					
Obesity	308 (66)	37 (74)	2260 (28)	1.49 (0.77−2.88)	**7.32 (3.88−13.80)**
Dyslipidemia	128 (27)	15 (30)	1022 (13)	1.14 (0.60−2.16)	**2.96 (1.61−5.43)**
Systemic hypertension	28 (6)	14 (28)	806 (10)	**6.13 (2.96−12.66)**	**3.51 (1.88−6.53)**

*SD* standard deviation, *IQR* interquartile range, *CI* confidence interval, *NASH* non-alcoholic steatohepatitis, *T2DM* type 2 diabetes mellitus

^a^NASH was defined as a child having ≥ 1 claims ICD-10 diagnosis code in the inpatient or emergency department setting or ≥ 2 claims diagnosis codes in the outpatient setting. T2DM was defined as a child having ≥ 1 claims ICD-10 diagnosis code in the inpatient or emergency department setting or ≥ 2 claims diagnosis codes in the outpatient setting or evidence of dispensed antidiabetic medication (except metformin and insulin alone) or metformin fill with presence of T2DM diagnosis code or insulin fill only with presence of T2DM or DM NOS

^b^Cells containing ten or fewer children are masked due to privacy rules

^c^Bolded values are statistically significant at p < .05

As shown in Table 2, children with NASH + T2DM had higher all-cause paid costs ($2,211 v. $663 per patient per month, p value < 0.0001) and more frequent all-cause hospitalizations (Incidence rate ratio = 2.4; 95% CI 1.3, 4.3, p value = 0.0038) compared to children with T2DM alone.

**Table 2:** Cost and healthcare utilization for childhood T2DM (Commercial and Medicaid) with and without NASH during follow-up period (after matching)

Measures	NASH + T2DM	T2DM only	Adjusted difference/Incidence rate ratio (IRR), (95% CI)^a^	P-Value^a^
Sample size, N	49	49		
Follow-up time, months, median (IQR)	19 (15)	21 (16)		
All-cause paid cost, PPPM, mean (SD)	$2,211 ($472)	$663 ($142)	**$1,549 ($561, $3,335)**	** < .0001**
All-cause paid cost, PPPM, median (IQR)	$576 ($1079)	$117 ($346)		
All-cause hospitalization, per 1,000 member-month, IR (95% CI)	39.4 (28.3, 54.9)	16.5 (10.1, 26.9)	**2.4 (1.3, 4.3)**	**0.0038**

Propensity score was calculated based on 1-year baseline values for age, Deyo-Charlson comorbidity index (DCI), adapted diabetes complications score, outpatient visits, office visits, ER visits, number of prescription classes dispensed, total medical cost, outpatient cost, inpatient cost, ER cost, gender, age group, DCI group, residence region, heart failure, obesity, hypertension, dyslipidemia, polycystic ovarian syndrome, hypothyroidism, sleep apnea, hypopituitarism, and hypogonadism

*PPPM* per patient per month which is a measure of actual paid cost that accounts for varying follow-up time for each child, *IR* incident ratio, *IRR* incident rate ratio, *SD* standard deviation, *IQR* Interquartile range, *CI* confidence interval, *NASH* non-alcoholic steatohepatitis, *T2DM* type 2 diabetes mellitus

^a^Bolded values are statistically significant at p < .05

## Discussion

In our study population, NASH was associated with significantly higher incremental cost and hospitalization among children with T2DM. However, diagnoses of NASH among children with T2DM appear very rarely. This differs from adults, where NASH is estimated to be present in up to 97% [2], although the point estimate is wide and varies depending on the population and measurement method. Given histological differences in pre-pubescent pediatric NASH liver biopsies compared to adults [3] and possible differences in genetic contributions in terms of susceptibility for progression to NASH [4], it is possible that NASH among children with T2DM represents variation of the underlying pathophysiological interplay between insulin resistance/hyperinsulinemia and liver disease compared to what is seen in adults. Alternately, our ascertainment of T2DM and NASH were claims-based, and data was not biopsy-verified NASH; furthermore, follow-up was limited to less than two years, on average. As such, there is possibility of misclassification, especially in the youngest children where T2DM is less prevalent.As recently recommended by the Liver Forum Pediatrics Working Group, emerging therapies for NASH should consider the needs for children with NASH as a distinct group from adults, including comorbid NASH and T2DM, which may constitute a special subgroup [5]. Our data provide meaningful real-world insights for clinicians, health systems, and payers regarding the incremental economic burden of NASH in children. As emerging therapies for NASH appear on the horizon, such analyses are instrumental in appropriately determining their overall value.

## Data Availability

Data from Medicaid and commercially insured members were combined for analysis. Only aggregate data was reported and no individual Medicaid states were identified or reported on. Medicaid state agencies that require approval of this publication have done so.

## Abbreviations

CI: Confidence interval
NASH: Non-alcoholic steatohepatitis
T2DM: Type 2 diabetes mellitus
